# The utility of low-cost negative pressure wound therapy in Cameroon

**DOI:** 10.11604/pamj.2024.48.151.39732

**Published:** 2024-08-05

**Authors:** Lahin Amlani, Henry Ndasi, Ghislain Aminake, Xavier Penda, Serge Timam, Aron Lechtig, Christina Barau Dejean, Kiran Agarwal-Harding

**Affiliations:** 1 Harvard Global Orthopaedics Collaborative, Boston, Massachusetts, United States of America,; 2 Johns Hopkins School of Medicine, Baltimore, Maryland, United States of America,; 3 Baptist Hospital Mutengene, Mutengene, Cameroon,; 4 Harvard Combined Orthopaedic Residency Program, Massachusetts General Hospital, Boston, Massachusetts, United States of America,; 5 Department of Orthopaedic Surgery, Hospital Universitaire La Paix, Port-au-Prince, Haiti,; 6 Department of Orthopaedic Surgery, Beth Israel Deaconess Medical Center, Boston, Massachusetts, United States of America

**Keywords:** Orthopaedics, surgery, trauma, health system development, medical device

## Abstract

Musculoskeletal injuries are common in Cameroon. Negative pressure wound therapy (NPWT) can effectively manage complex wounds including open fractures, however high cost and unavailability prevent its widespread use. We sought to evaluate the feasibility and efficacy in Cameroon of a low-cost NPWT (LCNPWT) device built from an aquarium pump costing less than $100. We performed a prospective case series including all patients with musculoskeletal injuries managed with LCNPWT at Baptist Hospital Mutengene, Mutengene, Cameroon from 15^th^ March 2021 to 15^th^ March 2022. Patient demographics, wound characteristics, and wound photographs were collected at intake and at each dressing change (performed every 3 days). All treatment was provided inpatient, and outcomes were recorded at hospital discharge. Forty-one patients (mean age 40 years, 58% male) received LCNPWT. The most common injury mechanisms were road traffic-related accidents (n=16, 42%) and gunshots (n=8, 21%). Wound characteristics were recorded for 38 patients of which 24 (63%) had infected wounds and 3 were bacteremic (13%) on presentation. All patients received antibiotics. The average duration of LCNPWT was 5.9 days (standard deviation 3.1 days). For 15 patients with documented outcome data, LCNPWT was successful in achieving wound closure in 12 (80%). Five patients failed limb salvage, with 4 receiving amputations and 1 dying during hospitalization. Eighty-three percent of providers (15 providers) reported LCNPWT as beneficial in managing wounds. Low-cost NPWT device was effective for managing contaminated and complex wounds in a resource-limited setting.

## Introduction

Trauma is a leading cause of mortality worldwide, greater than HIV/AIDS, tuberculosis, and malaria combined [1]. Death from trauma is disproportionately high and rising in low- and middle-income countries (LMICs) [2]. For every trauma-related death, many more suffer from morbidity and disability [3]. Cameroon is a lower-middle-income country located along the Atlantic coast of sub-Saharan Africa with a population of 25 million people [4]. Cameroon has the 23^rd^ highest road traffic mortality rate worldwide at 30 per 100,000 people [4]. Moreover, sectarian conflict and violence between anglophone separatist fighters and the government military in Western Cameroon, and between Boko Haram and counter-insurgency government forces in Northern Cameroon have increased the burden of trauma [5].

Negative pressure wound therapy (NPWT) has been widely accepted as adjuvant therapy across surgical subspecialties in the management of acute and chronic wounds of both adults and children [6]. In LMICs, the high costs associated with NPWT devices present a significant barrier to their use. In Cameroon where healthcare financing is out of pocket and the availability of NPWT devices is lacking, the feasibility and efficacy of affordable NPWT are unknown. Previous studies have shown the efficacy of low-cost NPWT devices in Haiti, the Philippines, and Brazil [7-9]. In a multinational collaboration between colleagues in Haiti, Cameroon, and the United States of America, we designed a modified low-cost NPWT (LCNPWT) suction pump based on the Turtle Vac described by Dejean *et al*. [7]. Similar to the Aquapump design described by Cocjin *et al*. [8], the LCNPWT device used in this study was made from an aquarium aerator pump (Tetra Whisper 100) powered by 110V AC, a pressure gauge, and a bleed valve to regulate pressure. Costing less than 100 USD in supplies, the LCNPWT device is a fraction of the cost of commercially available NPWT pumps, like the Wound VAC® by KCI/3M which costs $40,000 [10]. It can accommodate standard medical suction tubing and a variety of suction canisters. Wound dressings are typically made by using sterile gauze or sterilized locally available sponge material to fill the wound defect; and nonsterile 3M^TM^ Ioban^TM^ dressing, 3M^TM^ Tegaderm^TM^ dressing, or similar plastic adhesive film dressing to seal the wound. We sought to evaluate the feasibility and efficacy of LCNPWT in the resource-limited setting of Cameroon.

## Methods

**Study setting and population:** we performed a prospective case series from March 2021 to March 2022 at Baptist Hospital Mutengene, Mutengene, Cameroon. Patients were prospectively identified as candidates for NPWT and enrolled in the study by their treating orthopaedic surgeon. Full discretion regarding patient selection was left to Cameroonian surgeons to allow for seamless workflow integration and a better understanding of current provider decision-making processes in this low-resource setting. Low-cost NPWT (LCNPWT) was only offered to patients admitted as inpatients to standardize care and ensure consistent NPWT use. Patients were excluded from the study if they were not able to remain in the hospital for the duration of treatment; if amputation of the injured extremity was planned within 2 weeks of presentation; if the patient had known vasculopathies, autoimmune, or connective tissue disease; or the patient had a vasopressor requirement.

**Figure 1:**
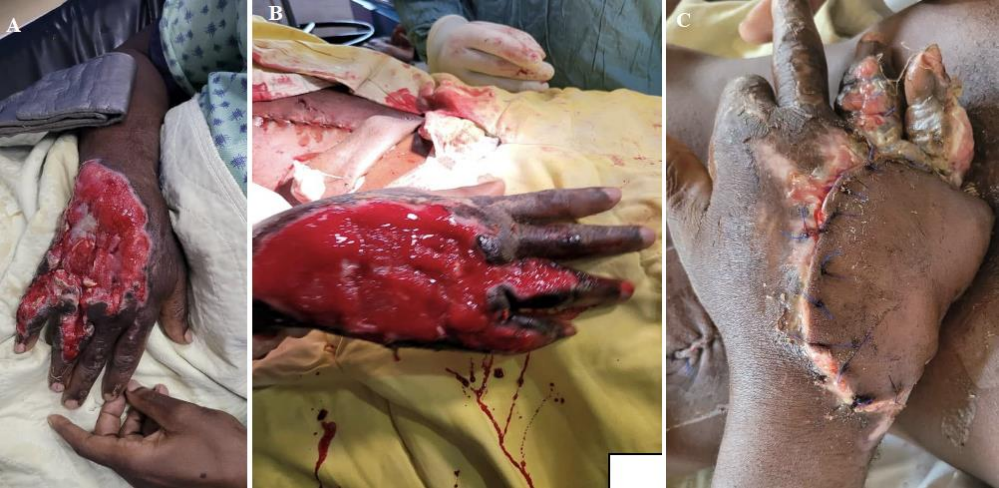
representative case of a dorsal hand wound at 3 stages of management: A) before; B) after LCNPWT; C) after flap coverage for definitive closure

**Figure 2:**
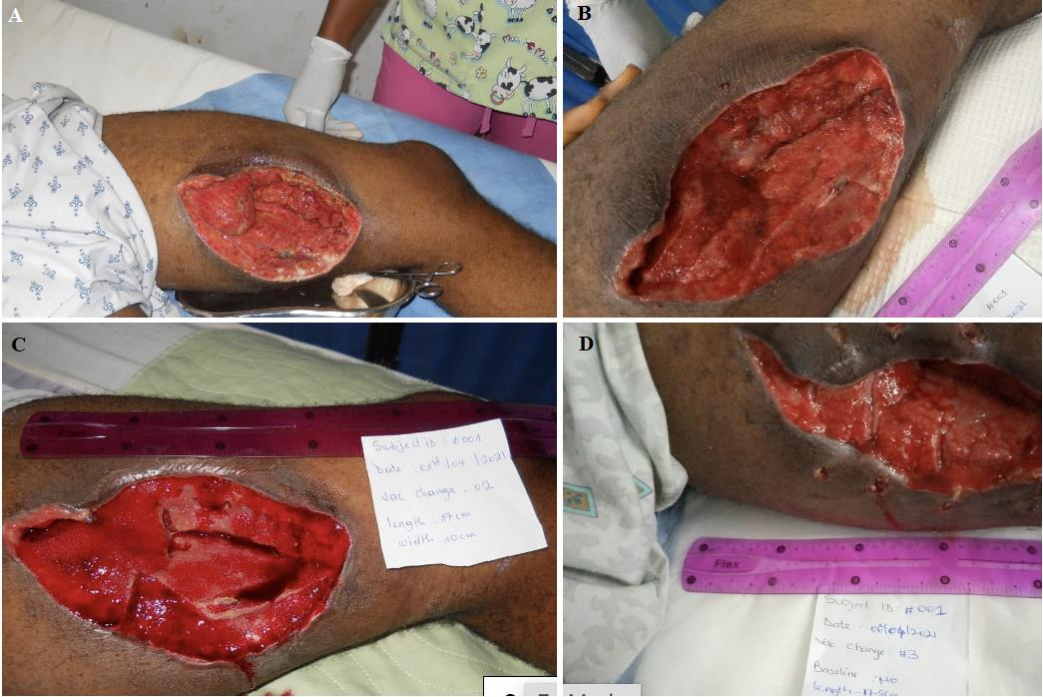
the representative case of a medial thigh wound at 4 stages of management with LCNPWT: A) before; (B,C,D) after three rounds of LCNPWT with the sequential reduction in wound size, healthy granulation tissue, allowing eventual primary closure

**Treatment technique:** wounds were first debrided and necrotic tissue was removed until actively bleeding tissues were seen. The wound was then measured and pre-LCNPWT photos were taken (Figure 1, Figure 2). A sterile wound sponge or gauze was placed to cover the entirety of the wound and the wound was then sealed with Ioban™, Tegaderm™, or a locally available equivalent plastic film dressing. A surgical suction tube was perforated with 2-3 holes approximately 1 cm apart along the same surface and inserted into the dressing through a perforation in the plastic film. Small strips of plastic film (approximately 5 x 7 cm) were used to reinforce the dressing around the tube to create an airtight seal. The tube was attached to a standard medical suction canister, then the suction canister was connected with standard suction tubing to the LCNPWT device. Following full assembly of the LCNPWT dressing, the LCNPWT device was plugged in, the sponge in the wound deflated, and the pressure gauge began to show negative pressure. Care was taken to ensure that pressure never exceeded -125 mm Hg. The patients were then transferred to the wards and any leaks in the seal were reinforced with plastic film dressing or tape at the bedside. Suction canisters were exchanged as needed to manage fluids. Dressings were changed every 3 days with new gauze/foam and plastic film applied, often with wound irrigation and debridement.

**Data collection methods and analysis:** following informed consent, patients were enrolled in the study and the treating surgeon recorded patient demographics, injury information, and wound characteristics. We photographed the wounds prior to the application of LCNPWT and recorded the supplies used, suction achieved, and the next scheduled dressing change. At each dressing change, we recorded the wound characteristics, photographed the wound, described the exudate, and recorded the suction achieved after dressing and any problems with the LCNPWT application. At the completion of treatment, we recorded the final outcome and any complications, including lack of granulation tissue formation, systemic infection, sepsis at any point during therapy, amputation, or any other reason for the failure of LCNPWT. Ethical approval for this study was provided by the Institutional Review Board (IRB) of the Cameroon Baptist Convention Health Services, and the Beth Israel Deaconess Medical Center Committee on Clinical Investigations gave approval for analysis of the de-identified dataset.

## Results

Forty-one patients were enrolled in the study, the median age was 40 (standard deviation, SD ± 14), and 58% were male. Seventeen (44%) patients were farmers, 5 (13%) were students, 4 (11%) were businesspeople, 4 (11%) were housewives, 3 (8%) were drivers, and 5 (13%) had some other occupation. The most common comorbidity among patients was alcohol consumption (23 patients, 60%), followed by smoking in the last 6 months (5 patients, 13%), diabetes (4 patients, 11%), and recreational drug use (3 patients, 26%). Ten (26%) patients had other medical conditions (Annex 1). The median travel time from home to the hospital was 2 hours (interquartile range (IQR) 1-4 hours). Patients presented to the hospital an average of 2 days (IQR 0-6 days) after injury (Table 1).

**Table 1:** demographic characteristics of study participants, enrolled for management of wounds with LCNPWT at Baptist Hospital Mutengene, Cameroon, March 2021 to March 2022 (N=41)

Age	40 (SD ± 14)
Male	24 (58%)
**Occupation (N=38)**	
Student	5 (13%)
Farmer	17 (44%)
Business	4 (11%)
Housewife	4 (11%)
Driver	3 (8%)
Other	5 (13%)
**Comorbidities (N=38)**	
Diabetes	4 (11%)
Smoke in the last 6 months?	5 (13%)
Alcohol	23 (60%)
Recreational drugs	3 (8%)
Any other medical condition	10 (26%)
Median travel time from hometown to hospital (hours) (N=36)	2 (IQR 1-4)
Median days presenting to hospital after injury (N=31)	2 days (IQR 0-6)

LCNPWT: low-cost negative pressure wound therapy; percentages may not add up to 100% due to rounding; number of patients in each category may vary based on available information. N values are specified if less than the total overall cohort of 41 patients

Road traffic injuries were the most common mechanism of injury (16 patients, 42%), followed by gunshot injuries (8 patients, 21%), fall from height (3 patients, 8%), and stabbing (1 patient, 3%). Ten (26%) patients had some other mechanism of injury (Annex 1). Injuries to the lower extremity were most common (35 patients, 92%), specifically the leg (16 patients, 42%), foot (7 patients, 18%), and pelvis (4 patients, 11). Most wounds were reported as infected (24 patients, 63%) by the treating providers, though no cultures were obtained. Thirty-six (95%) patients were given broad-spectrum antibiotics. Three (13%) patients were bacteremic on presentation. The median suction achieved after dressings were applied was 89 mmHg (IQR 76-102 mmHg). Three (19%) patients required serial irrigation and debridement. The median length of LCNPWT was 6 days (IQR 3-7 days) (Table 2).

**Table 2:** wound characteristics, location, and dressing change data of study participants, enrolled for management of wounds with LCNPWT at Baptist Hospital Mutengene, Cameroon, March 2021 to March 2022 (N=41)

Mechanism of injury	
Road traffic-related injuries	16 (42%)
Gunshot injuries	8 (21%)
Fall from height	3 (8%)
Stabbed	1 (3%)
Other	10 (26%)
Isolated Injury	1 (3%)
**Injury location**	
Leg	16 (42%)
Foot	7 (18%)
Pelvis	4 (11%)
Thigh	3 (8%)
Ankle	3 (8%)
Knee	2 (5%)
Torso	2 (5%)
Arm	1 (3%)
**Wound characteristics**	
Infected	24 (63%)
If infected, cultures?	0 (0%)
Antibiotics	36 (95%)
Bacteremia	3 (13%)
Frequency of vac changes (days) (median)	3 (IQR 3)
Suction after dressing applied (mm Hg) (median)	89 (IQR 76-102)
Need of serial I&D	3 (19%)
Duration of vac treatment (days) (median)	6 (IQR 3-7)

I&D: incision and drainage; LCNPWT: low-cost negative pressure wound therapy; percentages are calculated based on available responses, not the total number of patients in the study (N=41), and therefore may vary by category

Out of 53 dressing changes recorded, wound margins had improved in 47 (89%), were the same for 5 (9%), and worse for 1 (2%). Figure 1 and Figure 2 show representative wound progression during LCNPWT. Of 15 patients whose outcomes were recorded, 12 (80%) achieved wound closure of which 6 patients received skin grafting (Table 3). Four patients failed limb salvage and required amputation, and one patient died. Figure 1 displays a representative case from the case series, a wound on the dorsal right hand at 3 stages of management: immediately post debridement (Figure 1A), following the use of LCNPWT (Figure 1B), and following flap coverage (Figure 1C). Figure 2 shows representative wound progression during LCNPWT. Figure 2 shows another representative case of a wound on the medial aspect of the thigh at 4 stages of management: immediately post debridement (Figure 2A), after first LCNPWT application for 3 days (Figure 2B), after second application of LCNPWT with measurable decrease in wound size and further wound healing (Figure 2C), and after final 3-day application of LCNPWT with a notably smaller wound and healthy granulation tissue that was primarily closed (Figure 2D).

**Table 3:** wound outcome, patient outcome, and LCNPWT challenges reported for study participants, at Baptist Hospital Mutengene, Cameroon, March 2021 to March 2022 (N=41)

The margins look compared to the last change	
Better	47 (89%)
Same	5 (9%)
Worse	1 (2%)
**Overall, the wound looks compared to the last change**	
Better	44 (86%)
Same	5 (10%)
Worse	2 (4%)
Skin grafted? (Yes)	9 (50%)
Was wound closure achieved? (Yes)	12 (80%)
Did the patient develop a systemic infection from the wound? (Yes)	0 (0%)
Did the patient become septic at any point? (Yes)	0 (0%)
Was the limb amputated? (Yes)	4 (21%)
Did the patient expire? (Yes)	1 (5%)
Issues with LCNPWT pump (Yes)	11 (22%)
Sealing	9 (64%)
Pump	3 (21%)
Bottle	1 (7%)
Receiver	1 (7%)
Was NPWT effective in helping you manage the patient's wound? (Yes)	15 (83%)
Did you experience any challenges in using NPWT while caring for this patient? (Yes)	2 (11%)

LCNPWT: low-cost negative pressure wound therapy; challenges in using LCNWPT are reported by the treatment provided. Percentages are calculated based on available responses, not total number of patients in the study (N=41), and therefore may vary by category

Among the four patients who received amputation, 2 patients (50%) had diabetes, 1 patient (25%) used recreational drugs, and 1 patient (25%) had hypertension. One patient suffered from a gunshot wound (25%), one patient (25%) was stabbed, and 2 patients (50%) had an unknown source of injury. All four patients suffered severe wounds on the leg and refused primary amputation, though it was recommended to all four patients at the initial presentation. Low-cost NPWT (LCNPWT) was applied in an attempt at limb salvage but ultimately failed.

One patient, a 34-year-old male with hypertension, heart failure, and Cushing syndrome was treated with LCNPWT for wounds sustained to his back, abdomen, and gluteal muscles. Providers reported the NPWT was not effective in managing this patient´s wounds. The patient passed away one month after admission to the hospital.

During 51 dressing changes in which providers gave feedback regarding LCNPWT, providers reported problems with the LCNPWT system on 11 (22%) occasions. The most common problem was achieving adequate seal which occurred during 9 (18%) dressing changes. Low-cost NPWT (LCNPWT) device and suction canister were reported to cause problems during 3 (6%) and 2 (4%) dressing changes, respectively. Of the 18 patients for whom treatment completion data were recorded, providers reported that LCNPWT was effective in wound management for 15 (83%) patients.

## Discussion

Negative pressure wound therapy (NPWT) is a proven, effective adjuvant therapy in the management of wounds and open fractures [6]. In resource-limited settings like Cameroon, NPWT is unavailable because of the high cost of the devices and supplies. Low-cost methods of NPWT have been previously described using an aquarium pump and wall suction pressure stabilizer [7-9]. Therefore, our prospective case series sought to determine the feasibility and efficacy of low-cost NPWT in Cameroon, as well as identify the challenges with its use. We found that NPWT was primarily used in the management of lower extremity injuries and was an effective adjuvant treatment in the management of most wounds and open fractures according to providers. However, the median suction achieved was considerably lower than the established standard of care (-125 mmHg), and providers reported challenges with NPWT use mainly due to leaks in the dressing and inadequate single-use supplies.

Negative pressure wound therapy (NPWT) has demonstrated faster healing rates compared to conventional dressings [11]. Previous studies have shown the efficacy of commercially available NPWT systems in reducing complications from acute and chronic wounds [12,13]. NPWT promotes wound healing by providing a moist wound bed, reducing edema, removing healing inhibitors, increasing blood flow, promoting granulation tissue, controlling mechanical stress in the wound bed, and promoting cell proliferation [13]. NPWT can be used for wounds of any size, though it is contraindicated for critically ischemic wounds [13]. The high cost of NPWT devices and single-use, proprietary supplies has made NPWT inaccessible to many in low-resource settings. Multiple groups have demonstrated effective low-cost NPWT devices. Barau Dejean *et al*. documented the efficacy of the “Turtle VAC” in three patients in Haiti [7]. Cocjin *et al*. showed their AquaVAC to be non-inferior to commercially available NPWT in a series of 36 patients in the Philippines [8]. Similarly, a custom, wall-mounted NPWT device (USP) was shown to be non-inferior in a randomized control trial of 72 patients in Sao Paulo, Brazil [9]. Our LCNPWT device was an effective adjuvant treatment in the management of acute and chronic wounds in Cameroon. Four patients in our series failed limb salvage and received amputation, and one patient expired, likely due to many additional chronic and acute comorbid conditions.

**Limitations:** while we found LCNPWT to be effective, many challenges remained. First, the most common challenge reported using LCNPWT was difficulty achieving an adequate seal in the dressing. Locally available plastic film was used to create a seal around the wound, and 64% of issues with LCNPWT usage were caused from inadequate adhesive properties of this material. This challenge was overcome by using a medical-grade adhesive dressing in some cases, though this dressing material was considerably more expensive and was available only through external donations to the study sites. A consistent supply of medical-grade adhesive dressing should be further explored and will require the identification of low-cost adhesive dressing manufacturers and the establishment of a reliable supply chain. Alternatively, leaks in the dressing could be overcome by increasing the suction force of the pump. The LCNPWT devices used in this study had a maximum suction of approximately -200 mmHg (depending on the individual device), however, the leaks in the dressing meant that average suction remained below the standard -125 mmHg. Low-cost NPWT (LCNPWT) devices could be developed to achieve a higher maximum suction with pressure monitoring and adjustment to ensure that pressure is below -125 mmHg to avoid barotrauma. Second, although LCNPWT is low-cost, it remains cost-prohibitive for the poorest patients and hospitals. Donations and innovative business models that can sustainably finance LCNWPT are necessary for the poorest populations. Third, LCNPWT durability was not evaluated in this study. No failures of LCNPWT pumps were reported, and at the time of this writing, all pumps were still operational. However, the full lifecycle of the LCNPWT pump has not been established. Fourth, LCNWPT devices were built with aquarium pumps optimized for 110V power input. However, in some areas, only 220V was available which necessitated the use of converters that reduced the suction power of the pumps. There is a need for LCNPWT devices compatible with variable voltage inputs, including 220V, and durable enough to withstand voltage and current fluctuations which are common in many resource-limited settings [14]. Fifth, LCNWPT requires a consistent power source and could therefore only be used in the inpatient setting. Recently, battery-powered ultraportable NPWT devices have been introduced in high-income settings. These battery-powered devices have discrete dimensions and are easily transported. However, these devices can be limited in their suction capacity (some devices have a maximum suction of -80 mmHg), can only treat small, moderate wounds, and most only function for 7 to 15 days [15,16]. Mechanical pump devices require excellent dressing seals to maintain suction, which is challenging in most complex orthopaedic injuries which are often large and require dressings around external fixator pins [17]. Further development and cost-reduction are required before these devices can be considered as a pragmatic option as therapy for orthopaedic trauma in low-resources settings.

A major limitation of this study was many patients had incomplete paper data collection forms which, may have altered the results of the study. Moreover, our data were collected at one hospital in Cameroon and the results may not be generalizable to other hospitals in Cameroon or other countries. However, we do believe that the high burden of traumatic injuries and limited access to modern surgical tools observed in this study closely resembles the situation in many resource-limited hospitals in LMICs [1,18-20].

## Conclusion

Despite its limitations, this study demonstrates the feasibility and efficacy of low-cost NPWT in a resource-limited setting like Cameroon. NPWT is an important temporizing and adjuvant treatment for patients with acute and chronic complex wounds and open fractures. Unfortunately, this technology is unavailable to many of the world´s poorest patients, who often suffer the highest incidence of trauma. In Cameroon, as shown elsewhere, NPWT can be effective and affordable but challenges persist. Many LCNPWT pumps have been described, and to be effective in the resource-limited setting, they must be durable, effective, and safe, with adjustable suction to accommodate for leaks in the dressing and the ability to handle variable voltages and currents. Low-cost NPWT (LCNPWT) also requires a sustainable supply of effective dressing materials and a standardized method of treatment. Future implementation of LCNPWT in LMICs should emphasize the development of a system of care that incorporates the pump, dressings, staff training, and outcome measurement. Frugal innovation with the poorest patients in mind has enormous potential to lower the barriers to essential trauma care but must be approached systematically, with the goal of demonstrating safety, efficacy, affordability, and sustainability.

### What is known about this topic

- *Negative pressure wound therapy (NPWT) is a safe, effective adjuvant treatment for the management of acute and chronic wounds in adults and children;*
- *Due to high costs, NPWT is unavailable to many living in low-resource settings;*
- *Previous low-cost negative pressure wound therapy devices have shown efficacy in a limited number of patients*.

### What this study adds

- *Our LCNPWT device was clinically effective and affordable at USD $100 in a low-resource setting such as Cameroon;*
- *Achieving adequate suction due to leaks in the dressings for complex wounds remains a challenge for LCNPWT, with affordable solutions*.

## Annex

**Annex 1**: any other medical conditions (PDF-62 KB)
